# Fit, Female or Fifty–Is Cardiac Rehabilitation “Fit” for Purpose for All? A Systematic Review and Meta-Analysis With Meta-Regression

**DOI:** 10.3389/fcvm.2022.764882

**Published:** 2022-03-29

**Authors:** Martin Smith, Jessica Orchard, Andre La Gerche, Robyn Gallagher, Jane Fitzpatrick

**Affiliations:** ^1^Australasian College of Sport and Exercise Physicians, Melbourne, VIC, Australia; ^2^Agnes Ginges Centre for Molecular Cardiology, Centenary Institute and The University of Sydney, Sydney, NSW, Australia; ^3^Clinical Research Department, Baker Heart and Diabetes Institute, Melbourne, VIC, Australia; ^4^Faculty of Medicine and Health, The University of Sydney, Sydney, NSW, Australia; ^5^Department of Physiotherapy, Centre for Health, Exercise and Sports Medicine, Melbourne School of Health Sciences, Faculty of Medicine, Dentistry and Health Sciences, University of Melbourne, Carlton, VIC, Australia

**Keywords:** exercise, exercise prescription, cardiac rehabilitation, women, young, risk factors

## Abstract

**Aims:**

Cardiac rehabilitation (CR) is an evidence-based intervention promoting risk factor modification following coronary artery disease events but the relative benefits for patient subgroups is not clear. This review synthesizes the available evidence on the effectiveness of modern CR programs and determines outcomes for age, sex and prior level of fitness.

**Methods:**

MEDLINE, CINAHL, and EMBASE were examined for RCT and cohort studies involving exercise prescription or phase II or III CR following Myocardial Infarction (MI), Percutaneous Coronary Intervention (PCI) and cardiac surgery from January 2010 to February 2021. Outcomes assessed included peakVO_2_max, 6-min walk test and Metabolic Equivalent of Task. Meta-regression was used to determine CR impact for change in fitness and age and sex influences.

**Results:**

The mean age of study participants was 59.5 years and 82.7% were male. Females, younger people and those of average or above cardiorespiratory fitness were substantially under-represented in data and attendance, with 13% of study groups with a mean age <55 years. At entry, 73% were below average for fitness vs. age-matched normative values. Fitness improved across all groups following CR with no evidence of sex or age independently affecting outcomes.

**Conclusions:**

Modest improvements in fitness in all groups were shown, but the benefits of CR can be far greater. A modern, innovative approach to CR will likely lead to more substantial benefits. This may require a “Precision Medicine” model which tailors exercise prescription to different populations to ensure all CR participant's needs are met. This will ensure that CR is more flexible and accessible for all.

## Introduction

Cardiovascular disease (CVD) is a leading contributor to global morbidity and mortality, causing approximately one third of total deaths annually (1). Mortality rates in high-income countries range from 20 to 50% (2, 3), with the worldwide disease burden from CVD being approximately 20% (1).

Cardiac rehabilitation (CR) is a multidisciplinary, evidence-based intervention (4), composed of a multifaceted set of interventions that aim to address common, modifiable risk factors for coronary artery disease (5). CR programs include risk factor education, behavior change and psychological support, but perhaps most importantly, exercise (5). Modern CR programs including early moderate intensity aerobic exercise (6) or high intensity training (7, 8) are well validated, safe and encouraged in current CR guidelines (9, 10).

Most international exercise guidelines focus on aerobic training as part of CR (11). There are significant variations in the way this is prescribed with regards to frequency, intensity, duration and type, as well as the length of the programs and whether resistance training is included (11). Guidelines tend to be generalisable in order to be applied to all patients, with variation minimized in exercise prescription between different clinical conditions and patients (11).

Although coronary heart disease and myocardial infarction (MI) predominantly affects older adults, young men or women can suffer MI. This carries significant morbidity, psychological and financial effects and high disease-adjusted life year (DALY) burdens (12). The prevalence of the disease in men and women aged between 45 and 54 years in England in 2019 was 2.7% (13), although other studies have shown this figure to be as high as 4% in people aged ≤40 years (14). A study in Melbourne, Australia, found that 20% of MI admissions were younger than 55 years (15). Evidence points toward younger CR patients having a higher rate of non-participation and dropout in CR (16). Secondary prevention is of utmost importance, particularly in younger people. Mortality in young patients with MI is as high as 30% at 15-year follow-up (17). Also of note, over a 14-year time period from 2003 to 2017 in England, while prevalence of coronary heart disease decreased by 25–43% in age groups aged over 55 years, there was no change in prevalence in the 44–54 years age group (13).

Women represent 36.3% of AMI presentations (18). Studies have shown that significantly fewer women subsequently access CR than men (19, 20), and those who do are less likely to complete the full duration of the program (21). Ritchey et al. (4) found that <1 in 5 women who are offered CR attend available programs. Women are not only less likely to receive evidence-based management, including revascularisation, preventative medications and CR, but they also often have poorer long-term outcomes (22).

Boyer et al. (23) showed that the characteristics of acute myocardial infarction (AMI) patients was changing in their study between 2003 and 2008. Their data showed an increasing proportion of AMI patients represented the 45 to 65-year-old age group in both males and females.

With this growing evidence pointing toward an increasing proportion of comparatively younger cardiac patients, as well as a lack of uptake of CR in females and younger people, we hypothesized that a similar trend would also be seen in patients entering CR from an average or above average fitness level for their age.

This study aimed to examine the effectiveness of modern (since 2010). CR programs, but perhaps more importantly, whether they examined for benefit in an increasingly heterogenous group of patients regardless of age, sex and prior level of fitness. The study also sought to assess whether the demographics of cardiac rehabilitation participants is changing in tandem with the changes in demographics seen in sufferers of AMI.

## Methods

This study was performed according to the “PRISMA statement for reporting systematic reviews and meta-analyses of studies that evaluate health care interventions” (24).

### Search Strategy

The literature search was performed using the MEDLINE, CINAHL, and EMBASE databases from January 2010 to February 2021. Search terms included: acute coronary syndrome, myocardial ischemia, coronary artery disease, heart valve disease, cardiovascular disease, cardiac event, coronary artery bypass graft, percutaneous coronary intervention, angioplasty, stent, valve repair, valve prosthesis, valve replacement, cardiac surgery, cardiac catheterisation, cardiac rehabilitation, exercise training and secondary prevention. The full search strategy is included in the Supplementary Material.

### Inclusion/Exclusion Criteria

Studies were included if they satisfied the following criteria: (i) original research published in English, (ii) contemporary, that is, published after January 2010, (iii) study population was adults over 18 years, (iv) studied Phase II (after discharge from hospital) or phase III CR programs (supervised or unsupervised outpatient CR), (v) described CR programs post myocardial infarction, PCI or cardiac surgery. Exclusion criteria were: (i) studies using only Phase I CR (in-patient, post event), (ii) inadequately described the exercise/rehabilitation protocol used, (iii) had fewer than 10 participants.

### Study Selection

Two researchers (MS and JF) screened the searched articles by title and abstract and all studies meeting the inclusion criteria were reviewed by full text. Any issues with eligibility were resolved by discussion with a third researcher (JO).

### Data Extraction

Data were extracted from the studies onto an Excel (Microsoft Corporation, USA) spreadsheet for analysis. Data extracted included author, year of publication, country of study, study design, number of participants, age, sex ratios, indication for CR, level of fitness at entry measured by baseline cardiorespiratory function tests, supervision level, CR setting, phase of rehabilitation, exercise program type, intensity and duration, primary and secondary outcome measures and dropout rates. Assessment of study methodological quality (type of study, level of evidence, performance of a power analysis, treatment allocation, blinding, dropout rates and methodological flaws) was performed. Only studies with level of evidence IV or greater were included to minimize bias. This is shown in Table 1.

**Table 1:** Summary of study groups included within meta-analysis, with study types, key demographic data, CR delivery type and indication for CR. Level of evidence grading based on (25).

**References**	**Country of origin**	**Study design**	**No. of patients**	**Mean Age (SD)**	**Indication**	**% male**	**Supervision, Setting**	**Duration of CR (weeks)**	**Level of evidence**
**Phase 2**									
Malfatto et al. (26)	Italy	Prospective cohort	55	59 (10)	PCI after 1^st^ MI	81	S, OP	5	IV
Han et al. (27)	Taiwan	Prospective cohort	39	56.4 (10.8)	PCI	90	S, OP	12–26	IV
Pollman et al. (28)	Denmark	Retrospective cohort	146	63.5 (13)	Valve surgery	66	S, OP/IP	13	IV
Lee et al. (29)	South Korea	Prospective cohort	13	60.4 (5.2)	PCI	100	S, OP	12	IV
Baldasseroni et al. (30)	Italy	Prospective cohort	160	80.3 (4.2)	ACS or cardiac surgery	71	S, IP	4	IV
Andjic et al. (31)	Serbia	Prospective cohort	60	58 (8.4)	PCI	90	S, IP	3	IV
Kurose et al. (32)	Japan	Prospective cohort	41	63.1 (9.1)	Emergency PCI	100	S, not spec	26	IV
Madssen et al. (33) Aerobic interval	Norway	RCT	15	55.5 (50–60.5 95% CI)	PCI	93	S, OP	12	II
Madssen et al. (33) Moderate continuous	Norway	RCT	21	60.5 (56.5–63.5 95% CI)	PCI	71	S, OP	12	II
Pardaens et al. (34) Mitral valve, low risk	Belgium	Retrospective cohort	46	64 (9)	Mitral valve surgery	71	S, IP+OP	12–22	III
Pardaens et al. (34) Mitral valve, high risk	Belgium	Retrospective cohort	27	64 (9)	Mitral valve surgery	71	S, IP+OP	12–22	III
Pardaens et al. (34) Aortic valve, low risk	Belgium	Retrospective cohort	38	65 (12)	Aortic valve surgery	76	S, IP+OP	12-22	III
Pardaens et al. (34) Aortic valve, high risk	Belgium	Retrospective cohort	33	65 (12)	Aortic valve surgery	76	S, IP+OP	12–22	III
Keteyian, 2014– HIIT (35)	USA	RCT	15	60 (7)	CABG, PCI, MI	73	S, OP	10	II
Keteyian et al. (35) Moderate continuous	USA	RCT	13	58 (9)	CABG, PCI, MI	92	S, OP	10	II
Ortega et al. (36) supervised	Spain	RCT	46	Not spec	ACS, CABG, angioplasty	87	S, OP	≤18	II
Ortega et al. (36) unsupervised	Spain	RCT	51	Not spec	ACS, CABG, angioplasty	82	S, OP	≤18	II
Balsam et al. (37)	Poland	Prospective cohort	52	54.1 (7.1)	MI	89	S, OP	6	IV
Smialek et al. (38)	Poland	Prospective cohort	45	62.2 (range 24–81)	ICD insertion	62	S, IP+OP	20	IV
Bilinska et al. (39)	Poland	Prospective cohort	100	57 (6)	CABG	100	S, OP	6	IV
Moholdt et al. (40) residential	Norway	RCT	16	63.6 (7.3)	CABG	81	S, OP	4	II
Moholdt et al. (40) home-based	Norway	RCT	14	61.7 (8)	CABG	79	U, OP	4	II
Wu et al. (41)	Taiwan	RCT	61	59.6 (9.2)	CABG	82	S, OP	25	II
Temfemo et al. (42)	France	Prospective cohort	188	61.2 (13.4)	CABG, angioplasty, MI, valve replacement	60	S, OP	8	IV
Hsu et al. (43) CABG	Taiwan	Prospective cohort	34	57.2 (12.5)	CABG	79	S, OP	12	III
Hsu et al. (43) transplant	Taiwan	Prospective cohort	45	47.3 (14.5)	Heart transplant	80	S, OP	12	III
Gremeaux et al. (44) concentric	France	RCT	8	45.3 (5.2)	Stable CAD post PCI	100	S, OP	5	II
Gremeaux et al. (44) eccentric	France	RCT	7	53 (0.7)	Stable CAD post PCI	100	S, OP	5	II
Fang et al. (45) usual care	China	RCT	34	61.41 (10.2)	PCI	63	U, OP	Not specified	II
Fang et al. (45) home-based	China	RCT	33	60.24 (9.35)	PCI	63	U, OP	Not specified	II
Hayta and Korkmaz (46)	Turkey	Prospective cohort	56	57.21 (5.34)	CABG	Not specified	S, OP	12	IV
Huang et al. (47) short-term intensive	Taiwan	RCT	21	58.9 (3.1)	CABG	95	S, IP	4	II
Huang et al. (47) conventional	Taiwan	RCT	19	57.9 (1.2)	CABG	95	S, IP	4	II
Peixoto et al. (48)	Brazil	RCT	45	56.8 (10.8)	ACS and PCI	73	S, IP + U, OP	3	II
Wolszakiewicz et al. (49) interval	Poland	Prospective cohort	60	60 (7.8)	CABG	100	U, OP	3–4	III
Wolszakiewicz et al. (49) standard	Poland	Prospective cohort	59	56 (7.9)	CABG	100	U, OP	3–4	III
Laddu et al. (50)	Canada	Retrospective cohort	10,732	60.4 (10.5)	Cardiovascular disease	82	S, OP	Not specified	IV
Najafi and Nalini (51) home-based	Iran	Prospective cohort	195	54.53 (9.6)	Cardiovascular disease	79	S, IP/OP	13	III
Najafi and Nalini (51) hospital-based	Iran	Prospective cohort	585	55.89 (8.4)	Cardiovascular disease	75	S, IP/OP	13	III
Lee et al. (52) home-based	South Korea	RCT	26	54.3 (8.9)	PCI	85	U, OP	10	II
Lee et al. (52) usual care	South Korea	RCT	29	57.8 (7.5)	PCI	76	U, OP	10	II
Rechcinski et al. (53) incomplete revascularisation	Poland	Retrospective cohort	49	58 (10)	ACS and PCI	73	S, OP	3	III
Rechcinski et al. (53) complete revascularisation	Poland	Retrospective cohort	141	58 (9)	ACS and PCI	63	S, OP	3	III
Smith et al. (54) hospital	Canada	RCT	74	70.3 (8.26)	CABG	80	S, OP	26	II
Smith et al. (54) home-based	Canada	RCT	70	70.2 (10.7)	CABG	87	U, OP	26	II
Amorim et al. (55)	Portugal	Retrospective cohort	238	53.6 (9.5)	ACS	84	S, OP	8	IV
Kamakura et al. (56)	Japan	Prospective cohort	219	55 (7)	AMI	88	S, OP	12	IV
Cao et al. (57)	China	Prospective cohort	24	59.4 (2.4)	PCI	79	S, OP	8	IV
Golabchi et al. (58)	Iran	Prospective cohort	15	54.2 (9)	AMI	100	S, OP	8	IV
Vysoky et al. (59)	Czech Republic	Prospective cohort	106	60.4 (10.9)	ACS	85	S, OP	8	IV
Dos Santos et al. (60) CR+inspiratory muscle training	Brazil	Prospective cohort	12	55 (7)	CABG	66.7	S, OP	12	III
Dos Santos et al. (60) CR only	Brazil	Prospective cohort	12	56.6 (5.5)	CABG	75	S, OP	12	III
Ko et al. (61) short-term	China	Prospective cohort	108	60.1 (7.8)	PCI	100	S, OP	13	IV
**Phase 2 and 3**									
Spiroski et al. (62)	Serbia	Prospective cohort	54	57.72 (7.61)	CABG	93	S+U, OP	29	IV
Zhang et al. (63)	China	RCT	65	70.3 (10.7)	PCI for STEMI	91	U, OP	46	II
**Phase 3**									
Kim and So (64) Men	South Korea	Prospective cohort	114	58.29 (10.33)	PCI	79	U, OP	35	III
Kim and So (64) Women	South Korea	Prospective cohort	30	60.9 (9.32)	PCI	79	U, OP	25	III
Mameletzi et al. (65)	Greece	RCT	10	71.1 (6)	CABG or PCI post AMI	100	S, OP	30	II
Ma et al. (66)	China	Retrospective cohort	32	59.3 (7.2)	STEMI	56	S, OP	25	IV
Kraal et al. (67) centre-based	Netherlands	Prospective cohort	45	57.7 (8.7)	ACS, PCI or CABG	89	S, OP	12	III
Kraal et al. (67) home-based	Netherlands	Prospective cohort	45	60.5 (8.8)	ACS, PCI or CABG	89	U, OP	12	III
Ko et al. (61) long-term	China	Prospective cohort	85	59 (8.8)	PCI	100	S, OP	52	IV

*RCT, randomized control trial; S, supervised rehabilitation; U, unsupervised rehabilitation; OP, outpatient rehabilitation; IP, inpatient rehabilitation*.

### Statistical Analyses

Statistical analysis was performed using Stata software (Version 14.0). Data from studies using the same outcome measures peak VO2max, 6-min walk test (6MWT) and Metabolic Equivalent of Task (METs) was pooled and analyzed with 95% confidence intervals (CIs) reported to assess for change in cardiorespiratory fitness from baseline to exit from rehabilitation. Where studies compared two different CR methods rather than a control group, both exercise protocols were included separately in the analysis. Where studies utilized two or more cardiorespiratory outcome measures, all were included. Heterogeneity assessed using an *I*^2^ test and a fixed effects model was used. A meta-regression analysis was performed to determine the effect of other factors, specifically age and sex.

## Results

### Study Characteristics and Patient Characteristics

**Figure 1:**
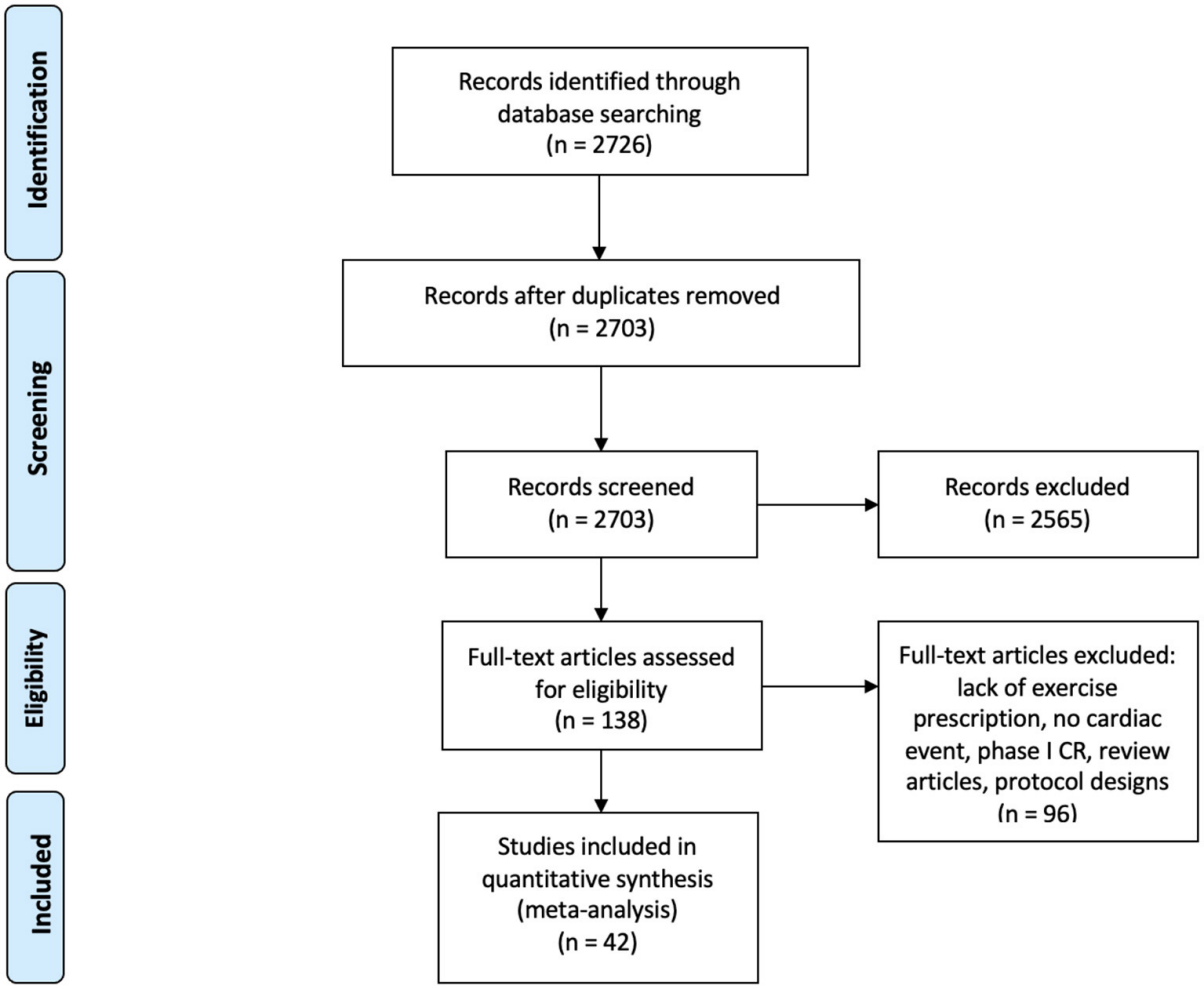
Preferred reporting items for systematic reviews and meta-analyses flow diagram of study selection.

The initial search identified 2,726 studies (Figure 1). A total of 23 duplicates were removed, and 2,565 records were excluded based on title and/or abstract screening, leaving 138 for full-text review. A further 96 articles were excluded due to lack of exercise prescription, no cardiac event, having only Phase I CR, no outcome measures to assess change in cardiorespiratory status, or were protocols or review articles. Thus 42 articles were available for quantitative analysis, of which 13 were randomized controlled trials (33, 35, 36, 40, 41, 44, 45, 47, 48, 52, 54, 63, 65) and 29 were cohort studies (26–32, 34, 37–39, 42, 43, 46, 49–51, 53, 55–62, 64, 66, 67). Included papers are shown in Table 1, together with study results and the level of evidence of the study. Studies were conducted primarily in Europe (21 studies) and Asia (16 studies) but also included Canada (2) and Brazil (2) and the USA (1).

A total of 14,683 patients were included in the study data. The mean age of study participants was 59.5 years (range of means 45.3–80.3 years). Eighteen studies included comparison of differing exercise protocols (including one that separated into four study groups), creating 62 groups of patients that completed CR. Of the study groups 8/60 (13%) had a mean age <55 years [one study encompassing two groups did not report mean age of participants (36)]. The mean percentage of male participants within the study groups was 82.7%. In total, 46/61 (75%) had ≥75% of participants being male (one study did not specify the sex breakdown of study participants).

Baseline level of fitness was examined by assessing values for peak VO_2max_, 6-minute walk test (6MWT) and METs on entry into CR, shown in Table 2. Mean baseline levels of cardiorespiratory function were: peakVO_2_ 23 ml/kg/min (age-based normal value = 25.1 ml/kg/min), 6MWT 420.5m (normal = 555) and baseline METs 7 (normal = 7). 27% of study groups had a mean average cardiorespiratory function level below age-matched normative values (68–70).

**Table 2:** Cardiorespiratory fitness levels on entry and exit from CR alongside normative values for community dwelling individuals aged 60–69 years.

		**Baseline**		**Exit from CR**	
	**Age-matched normative values (aged 60–69)**	**Mean**	**No. of study groups meeting normative values**	**Mean**	**No. of study groups meeting normative values**
Peak VO_2max_	25.1 (67)	23	27% (11/41)	26.5	54% (22/41)
6MWT	555 (68)	420.5	5% (1/22)	508.5	32% (7/22)
METs	7 (69)	7	69% (9/13)	8.6	77% (10/13)

*CR, cardiac rehabilitation; 6MWT, 6-min walk test; METs, metabolic equivalent of task*.

Peak VO_2_ was utilized by 28 studies (26–44, 56, 57, 59–62, 64, 65, 67), 12 studies examined 6MWT (34, 44–49, 60, 61, 63, 64, 66) and 9 studies utilized METs based on formal testing (50–55, 57–59). Multiple outcome measures were used in 7 studies (34, 44, 57, 59–61, 64). Dropout rate was specified in 16 of the studies (29, 33, 35, 36, 39–42, 44, 45, 51–54, 64, 65). The rate varied from 2 to 75%, with a mean dropout rate of 19.3%.

The duration of CR programs varied from 3 to 52 weeks, with a mean duration of 14.8 weeks. The prescribed programs are shown in Supplementary Table 1. The type of exercise prescribed varied, and included: walking (outdoor or treadmill), stationary bike, stationary stepping, stair climbing, swimming, arm ergometer, elliptical trainer, cross-country skiing, rowing and gymnastics. In some studies, strength training, calisthenics, or flexibility training were also included, but this was not examined in this study. Frequency of exercise was on average 3 sessions per week (range 2–10), with an average time of 38 min (range 10–90) min per session. Intensity levels varied between groups and were expressed as either: a percentage of VO_2_peak (5 groups, average 59–75% of VO_2_peak) a percentage of maximum or peak heart rate (11 groups, average 71–82% of maximum HR), a percentage of heart rate reserve (HRR) (13 groups, average 53.5–78% of HRR) or exercise at anaerobic or ventilatory threshold levels (6 groups). Nine study groups specified interval training, with the remainder focused on steady state training. Three studies were determined to be high-intensity interval training (HIIT) when defined as ≥85% of VO_2max_ (71).

**Figure 2:**
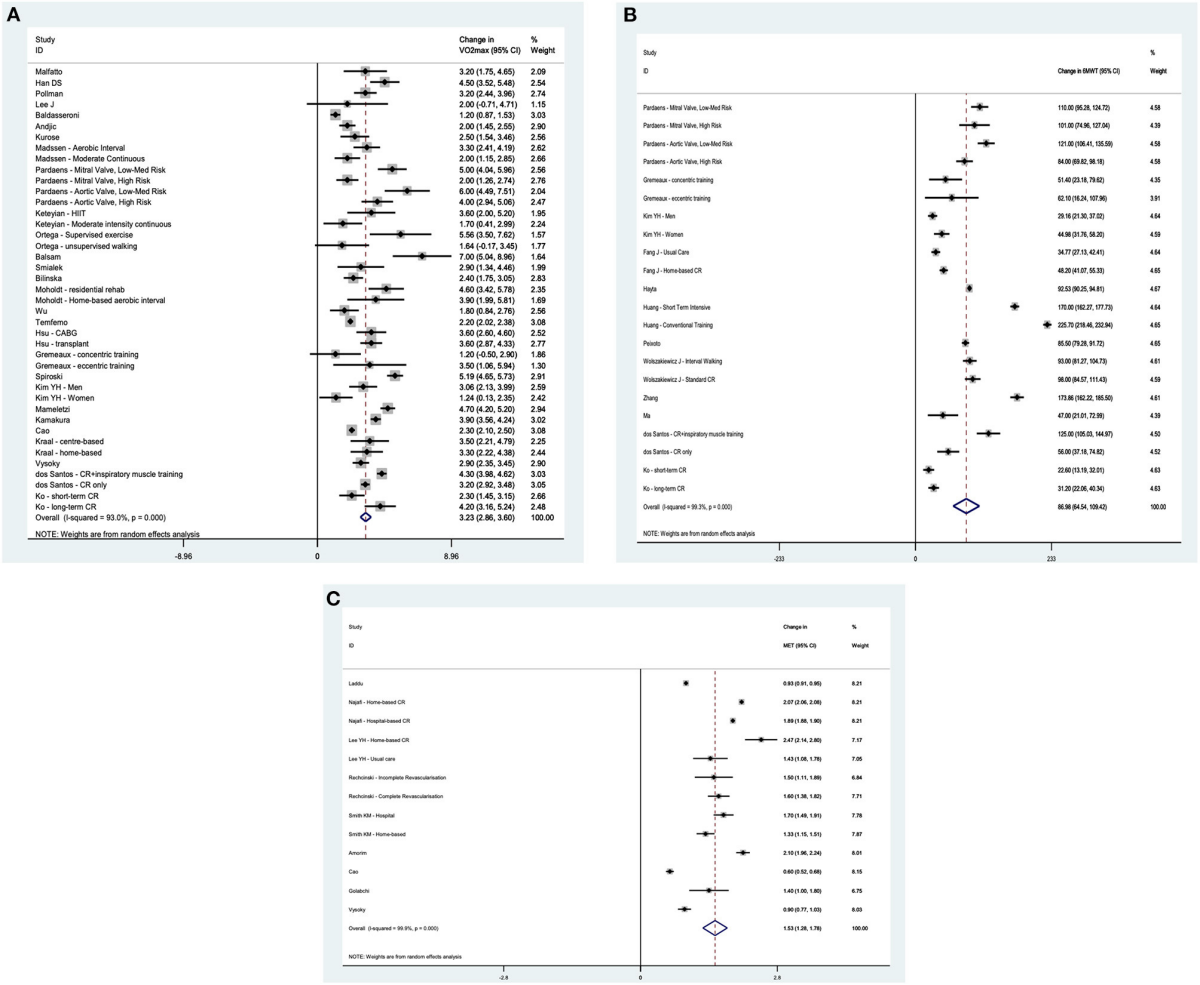
**(A)** Forest plot showing changes in VO2max in phase II and III cardiac rehabilitation programs. **(B)** Forest plot showing changes in 6MWT in phase II and III cardiac rehabilitation programs. **(C)** Forest plot showing changes in METs in phase II and III cardiac rehabilitation programs.

Cardiorespiratory fitness increased in all measures assessed across all study groups examined in this study. Change in peakVO_2max_ was assessed in a total of 2,141 patients, with an observed change of 3.23 ml/kg/min (2.86–3.60, 95% confidence interval). Mean change in 6MWT was examined in 999 patients, with a mean change of 86.98 m (64.54–109.42, 95% confidence interval) on exiting CR. The utilization of METs to assess change in fitness was used in 12,248 patients in total, showing a mean average improvement in METs of 1.53 METs (1.28–1.78, 95% confidence interval) from entry to exit from CR (Figure 2).

The proportion of patients meeting population age and sex referenced normative values for cardiorespiratory fitness increased across each of the three domains. On entry to CR, 27% (11/41) of study groups had a mean peakVO_2max_ at the level or above age-matched normative values. This increased to 54% (22/41) on exit from CR. Improvements were also seen in 6MWT from 5% (1/22 study groups) to 32% (7/22 study groups) and METs from 69% (9/13 study groups) to 77% (10/13 study groups).

A further meta-regression analysis was performed to assess the independent effects of age and sex on change in cardiorespiratory fitness with CR. For each outcome measure, age and proportion of male participants had no significant impact upon improvement in cardiorespiratory function following CR. The results of this analysis are shown in Table 3.

**Table 3:** Meta-regression analysis of the independent effects of age and sex on change in VO2max, 6MWT and METs following CR.

	**Male coefficient (95% confidence interval)**	**Age coefficient (95% confidence interval)**
VO_2max_	0 (−0.03, 0.41)	−0.02 (−0.09, 0.06)
6MWT	1.03 (−0.75, 2.80)	3.61 (−1.63, 8.85)
METs	0 (−0.05, 0.04)	−0.04 (−0.10, 0.03)

*CR, cardiac rehabilitation; 6MWT, 6-min walk test; METs, metabolic equivalent of task*.

## Discussion

This study presents a comprehensive review of the characteristics of modern CR attendees and study participants and the wide variations in how exercise is prescribed for patients. Our meta-analysis utilizes data from 42 studies, encompassing four continents over the past 11 years and involving 14,683 patients. Our results provide evidence that cardiac rehabilitation significantly improves the cardiorespiratory performance of attendees. It also demonstrates that within the dataset, the average CR patient is 60 years old, male and with low baseline cardiorespiratory fitness. The mean dropout rate in the studies examined in this review was 19.3%, which is concerning that we are potentially not meeting the needs for 1 in 5 patients. Whilst there is variability in the frequency, intensity, type and duration of CR programs, it may be that modern CR programs are focused for the mean average patient rather than tailored for individual needs.

### Sex

Lower uptake of CR in women compared to men has been identified in multiple studies (4, 19–21). Similarly, our study showed that only 17% of patients completing CR were female. This is despite 36.3% of patients suffering AMI being female (18), which would be represented the AMI and PCI aspects of this patient cohort. In addition, three-quarters of study groups had a male to female ratio of >3:1. Reasons previously attributed for this discrepancy have included: inadequate CR education and referral, transport and health system reasons, and competing demands (such as caregiver responsibilities) (72). However, even after age and covariate adjustment, women were found to be 9% less likely to attend CR and 13% less likely to complete CR after commencement (4).

Some efforts have been made to improve CR uptake and completion among female cardiac patients. One commonly used approach is to have sex-specific or women-only CR programs (22). This can lead to greater adherence (73), improved mental health outcomes (74) and comparable functional outcomes when compared to mixed sex CR (75), as well as improved satisfaction from participants (76). These classes more commonly offer alternative forms of exercise, such as dance and yoga. However, as an example in Australia, only 1% of programs offer women-only classes (22). Home-based or community-based programs have also been suggested as a potential improvement, as these have been shown to be equally effective as traditional CR (72) and may circumvent some of the potential barriers to attendance for women.

### Age

Previous studies have shown that younger age is associated with a higher drop-out rate (77). There is also evidence that older patients are more likely to complete CR (78, 79). This study appears to support these findings. The mean age of subjects completing CR in the groups analyzed was 59.5 years, but the average ages of study groups were closely centered around this point, showing little variability. Only 13% of study groups had a mean age <55 years, when this age group represents 23% of patients suffering AMI (80). As a result, when assessing the efficacy of CR programs, our data indicate that the research is skewed toward the needs of those aged older than 55.

In Australia and the UK, exercise recommendations for cardiac patients are largely toward light- to moderate-intensity aerobic exercise (81–83). Lower intensity exercise is easier to implement, particularly within restricted resource situations and is more acceptable to older adults and those with physical limitations (11), but this could be a significant deterrent to younger, more athletic patients, for whom the program is “too easy” (effectively detraining). Exercise interventions in coronary artery disease patients that recommend moderate to vigorous intensity exercise have shown greater improvements in cardiorespiratory fitness and cardiac risk factors than lower intensity interventions (11), as well as being safe and well accepted. Applying these measures to younger patients may lead to better uptake and completion, and better health outcomes.

### Fitness

Our study shows that the entry-level baseline level of fitness for patients completing CR was lower than that of normative data for sedentary 60–69 year-olds. We found that 73% of study groups were below average compared to their sedentary matched counterparts. That is, the majority of CR patients are below average fitness for their age.

It can be argued that acute events such as MI, PCI and cardiac surgery would be expected to acutely lower these cardiorespiratory functional measures. However, these low values may also represent drop-out or failure of inclusion of patients coming from a high level of baseline fitness capacity, and that these patients are disproportionately represented in the 19.3% of dropout cases from CR. Although this group may have lower risk for future cardiac events, the importance of transitioning them back to an acceptable level of function is high. A lack of guidance on how to progress back to normal function may increase the risk of further cardiac issues in this patient cohort.

### Exercise Prescription

The average CR program lasted 15 weeks and included cardiovascular training with 3 sessions per week. The average duration of each session was 38 min. Intensity levels were on average 59–75% of VO_2_peak, 71–82% of maximum HR, 53.5–78% of HRR or at anaerobic or ventilatory thresholds. Only 16% of groups were specified as interval training, with the remainder focused on steady state training. HIIT programs represented only 5% of prescribed programs.

Despite this study only including phase II or III CR programs, there was great variability in the programs prescribed to patients following cardiac events. This variability encompassed duration of programs, type of exercise program, frequency and duration of sessions as well as the intensity level and individualization of rehabilitation. This variation in prescription of exercise programs makes drawing conclusions for the type of rehabilitation that works optimally for different subsets of cardiac rehabilitation very difficult.

### Effectiveness of Modern CR

Cardiorespiratory fitness outcomes increased across all study groups examined in this study. Mean improvement in cardiorespiratory function was 3.23 ml/kg/min for VO_2max_, 86.98 m for 6MWT and 1.53 METs. The results of this study mirror those of a multitude of others showing that over the time period of CR, cardiorespiratory outcome measures improve on average in all participants.

Meta-regression analysis found that neither age nor the proportion of females to males in study groups had any significant impact upon improvement in cardiorespiratory function following CR. This highlights the importance of CR and its effectiveness for all patients, as well as the benefits that can be attained regardless of age and sex. However, it raises the question of whether this improvement could be increased further and whether rehabilitation is challenging enough or tailored enough to the patients involved.

### Survival of the (Not So) Fittest

This study displays the outcomes of CR in those who complete the rehabilitation process. This study compares the demographics of these patients to those eligible to complete CR and shows that younger, female and moderate-to-high fitness individuals are under-represented. CR appears to largely meet the needs of male, older and low fitness individuals, with either a failure to be referred, a failure to attend or a failure to complete rehabilitation in those outside of these demographics. It may be that due to rehabilitation being tailored to the more unfit members of the cohort, those of higher fitness feel that their needs are not met and drop out.

### Limitations

This study has a number of limitations. First, it examines CR research literature which includes both CR programs and research studies rather than a snapshot of CR programs themselves solely. Examining demographic trends within these data are beneficial and has been performed previously, but to our knowledge, no assessment of this demographic data has been performed on research study participants to this point. It is also important to reflect on the need for research looking at these specified groups and whether they benefit from rehabilitation as much as the more studied cohorts of males, sedentary and older patients.

Although this systematic review included all available literature, there were a small number of studies using each individual cardiorespiratory outcome measure resulting in high heterogeneity. Therefore, we have included only the three most widely reported outcome measures to minimize this heterogeneity. Additionally, regarding the delivery of cardiac rehabilitation, there is considerable variation particularly in the duration of cardiac rehabilitation. This varies greatly from study-to-study and country-to-country, leading to different effect sizes. The variation in the way that cardiac rehabilitation is prescribed and delivered in terms of frequency, intensity, type, and time, also means that there is unavoidable heterogeneity in results.

Most studies included did not specify baseline level of physical function and the level of physical activity prior to commencement of CR. Only 8/60 (13%) of studies specified a previous physical activity level. Baseline cardiorespiratory function testing was used for this purpose but may not adequately describe how physically active a rehabilitation participant was prior to commencement in CR. When grouped among other participants of low cardiac fitness, it will be difficult to ascertain whether the benefit gained from CR is greater or smaller than the mean for those who were previously physically active.

Dropout rate amongst studies was difficult to accurately determine as some studies only examined patients who completed CR whereas others included dropout rates from the commencement of CR. Whether these dropout rates preferentially affect younger, female and fitter patients is impossible to conclusively ascertain from this data. This is a potential area of future research, alongside further investigation into the reasons behind the poorer completion rate of each of these subgroups.

Cardiorespiratory assessment measures also appeared to be variable. In assessing baseline level of cardiorespiratory fitness, the percentage of study groups meeting age-matched normative values varied depending on which of the cardiorespiratory measures were used [peak VO_2max_ (27%), 6MWT (5%) and METs (69%)]. This may reflect inaccuracies in the normative values but may also show the inaccuracy of cardiorespiratory function testing in general, particularly with 6MWT and METs.

### Future Directions–From Efficacy to an Effective Intervention

This study highlights a number of concerns regarding the equity of CR. There appears to be a lack of research and exercise programs focused on the needs of female, young and fit patients when recovering from AMI, PCI or cardiac surgery. Taken together, this indicates large gaps in CR, with the possibility of patients “falling through the cracks” in recovering from a major life event. It also represents a potential risk of increased future cardiac events if the needs of these patients are not met.

In order to improve CR completion, it is important to address the reasons behind a lack of uptake and a lack of completion of CR in these patients. We need to ensure that CR is made more accessible and suitable to a broader range of people, as well as more appealing to those who are referred. By making CR more personalized, we can change CR from the current model of modest efficacy to an effective therapy.

Our opinion on how this is achieved is through a more patient-centric approach. Commencing any form of exercise is a two-stage process: motivation, as well as of performance. By addressing the goals, interests and motivations of the patient, greater long-term self-efficacy can be fostered. Moving away from the current paternalistic model of care does not have detrimental effects on patient outcomes and can be cost-effective, decrease fear and enhance motivation (45, 52, 84).

The future will involve a model of care that is more personalized and may be that a triage model is utilized involving clinical determinants of health as well as goals, risk factors and social determinants. Smart technology will allow a more specific, patient-centered approach, which can work better for those with time-constraints, whilst reducing stress and giving equivalent benefits to supervised training (45, 52). This can be done remotely and reach patients outside of suburban areas. Risk factors for coronary events appear to be changing, with a greater burden from sedentary behavior and lower physical fitness. By using more innovative methods to improve activity, risk factor modification can be sustainably improved.

## Conclusions

This study shows that there is a striking absence of females, younger people and those of average or above cardiorespiratory fitness in CR programs. As such, we do not currently understand the optimal method for prescribing exercise in rehabilitation for these patients and must do better at adapting methods for their needs, to make cardiac rehabilitation fit for purpose.

In this study, CR showed modest improvements in cardiorespiratory fitness for all demographic groups, but with a paucity of data in some subgroups. This leads us to believe that there is the potential for improvement in outcomes. A modern, innovative process will likely lead to greater benefits. This “Precision Medicine” model of exercise prescription may assist in this aim for improvement in secondary prevention goals for all participants, whilst making CR more flexible, more accessible and more easily scheduled into modern-day life. This tailored approach, with the inclusion of adapting programs to be specific to the needs, goals and enjoyment of participants, can lead to the integration of exercise and activity into daily life long-term, which is the most effective method of long-term prevention of cardiovascular morbidity and mortality.

## Data Availability Statement

The original contributions presented in the study are included in the article/Supplementary Material, further inquiries can be directed to the corresponding author.

## Author Contributions

MS, JO, and JF ran systematic review and assessed articles. MS, JO, JF, AL, and RG aided in writing various sections of the article. All authors contributed to the article and approved the submitted version.

## Funding

JO supported by a Postdoctoral Fellowship (Award Reference No. 104809) from the National Heart Foundation of Australia.

## Conflict of Interest

The authors declare that the research was conducted in the absence of any commercial or financial relationships that could be construed as a potential conflict of interest. The reviewer PQ declared a shared affiliation, with no collaboration, with one of the authors JO and RG to the handling editor at the time of the review.

## Publisher's Note

All claims expressed in this article are solely those of the authors and do not necessarily represent those of their affiliated organizations, or those of the publisher, the editors and the reviewers. Any product that may be evaluated in this article, or claim that may be made by its manufacturer, is not guaranteed or endorsed by the publisher.
