# Supplementary material for: Fit, Female or Fifty–Is Cardiac Rehabilitation “Fit” for Purpose for All? A Systematic Review and Meta-Analysis With Meta-Regression
Source: Front Cardiovasc Med. 2022 Mar 29;9:764882. doi: 10.3389/fcvm.2022.764882 (PMC9001939; doi:10.3389/fcvm.2022.764882)
Supplement: Supplementary file 1 [file Data_Sheet_1.pdf]

Search Strategy:

----

1 acute coronary syndrome.mp. or Acute Coronary Syndrome/

2 acs.mp.

3 myocardial ischemia.mp. or Myocardial Ischemia/

4 myocardial reperfusion injury.mp. or Myocardial Reperfusion Injury/

5 coronary disease.mp. or Coronary Artery Disease/ or Coronary Disease/

6 arteriosclerosis.mp. or Arteriosclerosis/

7 acute coronary syndrome.mp. or Acute Coronary Syndrome/

8 coronary occlusion.mp. or Coronary Occlusion/

9 heart valve diseases.mp. or Heart Valve Diseases/

10 Myocardial Infarction/ or acute myocardial infarction.mp.

11 MI.mp.

12 Cardiovascular Diseases/ or cardiovascular disease*.mp.

13 Heart Diseases/ or cardiac disease*.mp.

14 cardiac event*.mp.

15 1 or 2 or 3 or 4 or 5 or 6 or 7 or 8 or 9 or 10 or 11 or 12 or 13 or 14

16 Coronary Artery Bypass/ or coronary artery bypass graft.mp.

17 cabg.mp.

18 percutaneous coronary intervention.mp. or Percutaneous Coronary Intervention/

19 pci.mp.

20 percutaneous transluminal coronary angioplasty.mp. or Angioplasty, Balloon, Coronary/

21 Angioplasty, Balloon/ or Angioplasty, Laser/ or Angioplasty/ or Angioplasty, Balloon, Laser-Assisted/ or angioplasty.mp.

22 balloon angioplasty.mp.

23 laser angioplasty.mp.

24 Stents/ or coronary stent*.mp.

25 intracoronary stent.mp.

26 valve repair*.mp.

27 Heart Valve Prosthesis Implantation/ or Heart Valve Prosthesis/ or valve replacement.mp.

28 valve surgery.mp.

29 cardiac surgery.mp. or Thoracic Surgery/

30 Cardiac Surgical Procedures/ or cardiac surgical procedure*.mp.

31 cardiac catheterization.mp. or Cardiac Catheterization/

32 cardiac catheterisation.mp.

33 Transcatheter Aortic Valve Replacement/ or TAVI.mp.

34 16 or 17 or 18 or 19 or 20 or 21 or 22 or 23 or 24 or 25 or 26 or 27 or 28 or 29 or 30 or 31 or 32 or 33

35 cardiovascular rehabilitation.mp. or Cardiac Rehabilitation/

36 physical activity.mp. or Exercise/

37 exercise training.mp.

38 exercise therapy.mp. or Exercise Therapy/
39 strength rehabilitation.mp.
40 endurance activity.mp. or Physical Endurance/
41 cardiac rehabilitation.mp.
42 secondary prevention.mp. or Secondary Prevention/
43 15 and 34
44 exercise prescription.mp.
45 35 or 36 or 37 or 38 or 39 or 40 or 41 or 42 or 44
46 43 and 45
47 limit 46 to "all adult (19 plus years)"
48 limit 47 to dt=20200507-20210215
