# Supplementary material for: Fit, Female or Fifty–Is Cardiac Rehabilitation “Fit” for Purpose for All? A Systematic Review and Meta-Analysis With Meta-Regression
Source: Front Cardiovasc Med. 2022 Mar 29;9:764882. doi: 10.3389/fcvm.2022.764882 (PMC9001939; doi:10.3389/fcvm.2022.764882)
Supplement: Supplementary file 2 [file Table_1.pdf]

Supplementary Table 1 - Exercise protocols used within study groups, HR = heart rate, HIIT = high intensity interval training, AT = anaerobic threshold, HRR = heart rate reserve

Author, Year	Frequency (x/week)	Intensity	Borg Limits	Time (mins)	Type
**<u>Phase 2</u>**					
Malfatto, 2017 (38)	5	AT	-	65	Calisthenics and bike/treadmill
Han DS, 2017 (56)	2	55-70% VO2peak	12-13	30	Static bike
Pollman, 2017 (39)	2	60-80% VO2peak	-	90	Aerobic interval with resistance training
Lee J, 2017 (40)	2	70-85% max HR	-	40	Treadmill
Baldasseroni, 2016 (41)	5	60-70% VO2peak	11-13	30	Bike/calisthenics
Andjic, 2016 (42)	7	60-80% VO2peak	-	30	Intervals on bike
Kurose, 2016 (43)	3	AT	-	30	Bike/treadmill
Madssen, 2014 – Aerobic interval (25)	3	85-95% peak HR for intervals	-	28	Intervals on treadmill
Madssen, 2014 – Moderate continuous (25)	3	70% max HR	-	46	Treadmill
Pardaens, 2014 – Mitral valve, low risk (57)	2	AT	-	60	Cycling, walking and stepping
Pardaens, 2014 – Mitral valve, high risk (57)	2	AT	-	60	Cycling, walking and stepping
Pardaens, 2014 – Aortic valve, low risk (57)	2	AT	-	60	Cycling, walking and stepping
Pardaens, 2014 – Aortic valve, high risk (57)	2	AT	-	60	Cycling, walking and stepping
Keteyian, 2014– HIIT (26)	3	80-90% HRR	-	31	Intervals on Treadmill
Keteyian, 2014 – Moderate continuous (26)	3	60-80% HRR	-	30	Treadmill
Ortega, 2014 – supervised (27)	3-5	50-85% HRR	-	27	Static bike
Ortega, 2014 – unsupervised (27)	3-7	-	-	30-60	Walking
Balsam, 2013 (44)	3-5	HRrest+50% HRR	-	30	Bike

Smialek, 2013 (45)	5	40-60% max HR or HRrest + 10-30% HRR		10-30	Interval endurance
Bilinska, 2013 (46)	3	70-80% max HR	-	60	Bike intervals
Moholdt, 2012 – residential (28)	7.5	-	11-17	-	Outdoor walking, cross-country skiing, indoor cycling, strength training
Moholdt, 2012 – home-based (28)	3	85-95% max HR	-	28	Walking, jogging, swimming or cycling
Wu, 2012 (36)	3	60% HRR	-	30	Treadmill
Temfemo, 2011 (47)	3	Ventilatory threshold	-	45	Bike ergometer
Hsu – CABG (48)	3	50-80% VO2peak	-	25	Bike ergometer or treadmill
Hsu – transplant (48)	3	50-80% VO2peak	-	25	Bike ergometer or treadmill
Gremeaux – concentric (29)	3	Ventilatory threshold	-	90	Bike and arm cycling
Gremeaux – eccentric (29)	3	Ventilatory threshold	-	90	Bike and arm cycling – eccentric fashion
Fang – usual care (30)	2	General exercise advice	-	-	Patient preference
Fang – home-based (30)	3	General exercise advice	-	-	Outdoor walking or jogging
Hayta (49)	3	Not specified	-	30-50	Fast walking, low intensity running
Huang – short-term intensive (31)	3-8	80-120% ventilatory threshold	-	30	Stationary bike
Huang – conventional (31)	2-4	Not specified	-	20	Not specified
Peixoto (32)	4	-	4-5/10	20-40	Walking
Wolszakiewicz – interval (50)	5-6	Not specified	-	30-60	Isometric strengthening, general conditioning and bike ergometer or treadmill - intervals
Wolszakiewicz – standard (50)	5-6	Not specified	-	30-60	Isometric strengthening, general conditioning and bike ergometer or treadmill
Laddu (51)	4	45-85% HRR	12-14	60	Walking/elliptical/bike ergometer
Najafi – home-based (52)	5	Not specified	-	45	Dynamic exercise
Najafi – hospital-based (52)	5	Not specified	-	45	Dynamic exercise

Lee YH – home-based (37)	4-5	40-80% HRR		50	Gait exercise and flexibility
Lee YH – usual care (37)	-	Not specified	-	Not specified	General exercise advice
Rechcinski – incomplete revascularisation (53)	6	Not specified	-	10	Bike ergometer and resistance exercises
Rechcinski – complete revascularisation (53)	6	Not specified	-	10	Bike ergometer and resistance exercises
Smith KM – hospital (33)	3	60-80% HRR	-	30-50	Treadmill walking, stationary bike, arm ergometer, stair climbing
Smith KM – home-based (33)	3	60-80% HRR	-	30-50	Predominantly walking
Amorim (58)	2	60-80% HRR	11-13	35	Bike ergometer/treadmill
Kamakura (59)	3	50-60% HRR	12-13	45	Walking, bike or calisthenics
Ko (66)	3	40-70% HRR	-	30	Patient choice
Cao (60)	3	70-80% peak HR	-	30	Bike ergometer
Golabchi (63)	3	60-85% maxHR	-	30	Walking
**Phase 2 and 3**					
Spiroski (54)	8	70-85% max HR	-	30	Interval – bike and walking
Zhang (34)	3	60-75% max HR	12-16	30	Interval training
**Phase 3**					
Kim YH – Men (55)	3	40-75% HRR	-	30	Bike/treadmill
Kim YH – Women (55)	3	40-75% HRR	-	30	Bike/treadmill
Mameletzi (35)	3	70% max HR	13-14	30	Treadmill, bike, rowing, gymnastics
Kraal (62)	2	70-85% maxHR	-	45	Bike ergometer

Supplementary Table 1 - Exercise protocols used within study groups, HR = heart rate, HIIT = high intensity interval training, AT = anaerobic threshold, HRR = heart rate reserve
